# Differential correlations of serum PFASs with thyroid hormones in girls with central and incomplete precocious puberty: a machine learning analysis

**DOI:** 10.3389/fmolb.2026.1884470

**Published:** 2026-07-22

**Authors:** Yan Li, Yanan Xing, Xiaoqin Yin, Shaolian Zang, Ting Dong, Jiaoru Yang, Jiayin Dai, Yitao Pan, Nan Sheng, Pin Li

**Affiliations:** 1 Department of Endocrinology, Shanghai Children’s Hospital, School of Medicine, Shanghai Jiao Tong University, Shanghai, China; 2 Key Laboratory of Environmental Health Impact Assessment of Emerging Contaminants, Ministry of Ecology and Environment, School of Environmental Science and Engineering, Shanghai Jiao Tong University, Shanghai, China; 3 Key Laboratory of Environmental Health Impact Assessment of Emerging Contaminants, Ministry of Ecology and Environment, Shanghai Academy of Environmental Sciences, Shanghai, China; 4 Department of Urology, Shanghai Children’s Hospital, School of Medicine, Shanghai Jiao Tong University, Shanghai, China

**Keywords:** machine learning, PFASs, PFOA, PFBS, precocious puberty, thyroid hormones

## Abstract

**Background:**

Per- and polyfluoroalkyl substances (PFASs) are persistent environmental contaminants widely detected in Chinese children. Exposure has been associated with thyroid function and altered puberty timing.

**Objectives:**

To evaluate associations between serum PFAS concentrations and thyroid hormone (TH) levels in girls with central precocious puberty (CPP), incomplete precocious puberty (IPP), and healthy controls, and to identify key PFAS contributors using machine learning.

**Methods:**

In this prospective study, 943 serum samples were collected from 226 CPP, 316 IPP, and 401 healthy girls. Levels of 25 PFASs, total triiodothyronine (T3), thyroxine (T4), and reverse T3 (rT3) were measured. Multiple linear regression and random forest models assessed PFAS–TH associations. Feature importance (%IncMSE) was determined by random forest. Logistic regression evaluated odds of CPP and IPP associated with TH levels and interquartile shifts in key PFASs.

**Results:**

Among 25 PFASs, 12 had detection rates >95% and were analyzed. Perfluorooctanoic acid (PFOA) showed the highest levels, followed by perfluorooctane sulfonate. After adjustment, higher T3 was associated with increased odds of CPP (OR = 2.86) and IPP (OR = 6.38). In the CPP group, PFHpA was negatively associated with T3, while PFOA, Perfluorobutanesulfonic acid (PFBS), and Perfluorohexanesulfonic acid (PFHxS) were inversely associated with T4. In the IPP group, PFBS showed positive association with T3 but negative with T4; PFOS was also inversely associated with T4. The highest PFOA quartile linked to a 4.50% T4 decrease in CPP girls. PFBS at 0.20–0.58 ng/mL correlated with a 6.29% T4 reduction in the IPP group. Random forest explained 28.5%–35.4% of T4 variance across groups, with highest predictive power in IPP. PFOA and PFBS emerged as most influential for thyroid disruption (mean %IncMSE 21.9% and 22.2%).

**Conclusion:**

PFAS exposure and TH levels differ among CPP, IPP, and healthy girls, with concentration-dependent associations. PFOA and PFBS are key thyroid disruptors, and IPP girls appear particularly sensitive. Mechanistic studies are needed.

## 1 Introduction

PFASs are a collection of more than 4000 highly fluorinated aliphatic compounds employed in various uses (Sunderland et al., 2019). PFASs are extensively utilized in consumer goods like cookware, disposable food packaging, outdoor gear, furniture, and carpets due to their outstanding surface activity and stability. They are also one of the main components of aqueous film-forming foams used commonly at airports and by armies for firefighting and military training (Vecitis et al., 2010; Wang et al., 2017). Population exposure to PFASs occurs when individuals ingest polluted drinking water and seafood, inhale polluted air, and contact with other polluted materials (Trudel et al., 2008). Information on PFASs, such as PFOS and PFOA, in human sera, has been reported since 1990 (Hansen et al., 2001). As a result of phasing out certain products, many populations have seen a successful reduction in PFOS and PFOA exposure, but there has been an upward trend in exposure to C9–C11 perfluoroalkyl carboxylic acids (PFCAs) (Dassuncao et al., 2018). Several characteristics of health consequences, such as immune dysfunction, infection, asthma, cardiometabolic, neurodevelopmental, thyroid dysfunction, renal diseases, and puberty onset, have been observed.

Puberty is an essential, complex process involving genetic, nutritional, and environmental interactions (Subspecialty Group of Endocrinologic, H.t.S.o.P.C.M.A. Metabolic Diseases, and Editorial Board, C.J.o.P., 2015). An increasing number of children, especially girls, are experiencing earlier development of secondary sexual characteristics than has been considered normal (Ma et al., 2009). Environmental pollutants like endocrine disrupting chemicals (EDCs) have been linked to early puberty, as they can affect brain development, shift the timing of puberty, and lead to long-term reproductive effects. Research has concentrated on how PFASs impact neuroendocrine regulation of reproduction, the timing of puberty, and the female ovulation cycle in humans (Lopez-Rodriguez et al., 2021).

In childhood, thyroid hormones (THs) are essential for regulating energy expenditure, promoting growth, and supporting neurodevelopment. (Brent, 2012). Reports have shown that the pathways involved in the thyroid-disrupting effect are affected by PFASs (Ghassabian and Trasande, 2018). These mechanisms comprise a competitive process that impairs iodine uptake by thyroid cells and directly inhibits the NIS; hampers the creation of thyroglobulin; modifies the activity of TPO; disrupts feedback mechanisms or the biological effects of THs by altering the TH binding proteins (Ghassabian and Trasande, 2018).

In the literature, the thyroid-disrupting effects of EDCs were primarily associated with other EDCs rather than PFASs, and mainly observed in the general population or among mothers and newborns. This study aimed to investigate the possible association between background exposure to PFASs and serum THs levels in three groups: Chinese girls (7–10 years) with and without CPP and IPP.

## 2 Materials and methods

### 2.1 Sample collection

Between January 2021 and January 2022, 943 girls were recruited for this study: 226 girls diagnosed with CPP, 316 girls with IPP and 401 healthy girls. All girls were from the Shanghai Children’s Hospital. Inclusion criteria for CPP case subjects: (1) girls who underwent a physical examination, laboratory examination, evaluation of bone age, and transabdominal pelvic ultrasound, eventually diagnosed by a professional pediatrician; (2) girls with initial onset cases who never received medication. Exclusion criteria were as follows: (1) CPP of organic etiology or secondary to other diseases (e.g., McCune-Albright Syndrome and congenital hypothyroidism); (2) had received medication; (3) any chronic diseases, such as chronic abdominal distension, constipation, dyspepsia, and other gastrointestinal diseases and impaired liver function. IPP cases were restricted to girls with only breast development (isolated thelarche) or pubic hair (isolated pubarche). Girls with precocious puberty who had advanced height and bone age, enlarged uterine size, and LH levels exceeding 0.2 mU/L were not included (Banerjee and Bajpai, 2023). The girls in the control group were in good health and had no chronic diseases. Girls with secondary sexual development were excluded from the control group.

Shanghai Children’s Hospital’s Ethics Committee authorized this study. Written informed consent was obtained from participants or their legal guardian. The girls in the cases and controls were residents of Shanghai, Jiangsu Province, and Zhejiang Province.

### 2.2 Physical and laboratory examination

The following data were collected for each girl: age, height, weight, age at onset of secondary sexual characteristics (e.g., breast growth, pubic and axillary hair), age at menarche (if the girl has ever experienced menarche), tobacco smoking status of either parent of the girl, age of mother at menarche, age of mother at delivery, and mode of delivery.

Eligibility criteria for the CPP group included (Subspecialty Group of Endocrinologic, H.t.S.o.P.C.M.A. Metabolic Diseases, and Editorial Board, C.J.o.P., 2015) (1) onset of secondary sex characteristics before the chronological age (CA) of 8 years, including breast growth, pubic hair and axillary hair growth, or menarche before the CA of 10 years; (2) acceleration of linear growth: an annual growth velocity higher than that of normal children; (3) advanced bone age (BA)≥1 year above the CA; (4) transabdominal pelvic ultrasound scans: ovarian volume > 1.0–3.0 mL, with follicles larger than 4 mm; (5) based luteinizing hormone (LH) levels ≥ 3–3.5 IU/L; or LH peak levels ≥ 5 IU/L and LH/FSH >0.6 by GnRH provocation test.

The sexual development of all participants was evaluated by pediatric endocrinology physicians at the Shanghai Children’s Hospital. The Tanner method was used for pubertal staging (Marshall and Tanner, 1969). Patient weight and height were measured using the same apparatus in the outpatient clinic and during the same time of day (Kuczmarski et al., 2002; Ji and C. Working Group on Obesity in, 2005). Maternal age at menarche was classified as <13 years and ≥13 years (Meng et al., 2017). Using Greulich and Pyle’s standard method, bone age was determined from left hand and wrist radiographs (Subramanian and Viswanathan, 2022). Transabdominal pelvic ultrasound scans were conducted with a conventional 5 MHz scanner requiring a full bladder.

Prior to sample collection, all tubes and blood-sampling devices were verified to be free of perfluorinated compounds. Approximately 2 mL of blood was drawn from each participant, centrifuged at 3000 rpm for 15 min, and the resulting serum was stored at −80 °C until analysis. Hormones (LH and FSH) were measured by a solid-phase, two-site chemiluminescent immunometric assay on a Unicel Dxl 800 analyzer. Steroids were extracted with methanol, purified by solid-phase extraction using Waters Oasis PRiME HLB cartridges, and analyzed by UPLC-MS/MS on an Acquity I-class system equipped with a Waters ACQUITY UPLC BEH C8 column (1.7 μm, 100 mm × 2.1 mm) at 50 °C, coupled to a Xevo triple quadrupole mass spectrometer with ESI in both positive and negative modes (Peersman et al., 2020). T3, T4, rT3, and PFASs were measured by high performance liquid chromatography-tandem mass spectrometry (HPLC-MS/MS); 18 PFASs with detection rates >60% were included in the statistical analysis.

### 2.3 Statistical analysis

The representation of categorical variables involved numbers and percentages, and continuous variables were described using frequencies or means (standard deviation, SD). The limit of quantification (LOQ) divided by the square root of two was used as a replacement for concentrations that fell below the LOQ. The data of PFASs levels were ln-transformed to improve a normal distribution because of its skewed distribution, and T3, T4, and rT3 were normally distributed and not transformed. We used the t-test to examine differences in TH concentrations among the groups. Next, adjusted and unadjusted logistic regressions were applied to evaluate the THs levels in the CPP and control groups. Subsequently, using multiple linear regression, we analyzed the link between PFASs and THs for individual PFAS congeners and their sums in the CPP and control groups.

To assess multicollinearity among the 12 PFASs, we calculated variance inflation factors (VIFs) based on linear regression models. To further explore the effects of PFASs mixtures on THs and identify key pollutants, we employed a machine learning approach (random forest model). This model can automatically handle collinearity and nonlinear relationships, and provide feature importance rankings. For each group (control group, CPP group, IPP group), we separately constructed random forest models to predict T3, T4, and rT3, with the predictor variables being 12 PFASs with detection rates >95% (PFHpA, PFOA, PFNA, PFDA, PFUdA, PFDoA, PFBS, PFHxS, PFHpS, PFOS, 6:2Cl-PFESA, 8:2Cl-PFESA). The random forest model used 500 decision trees, with hyperparameters (mtry and node size) tuned via five-fold cross-validation. The final parameters were mtry = 4 and node size = 5. The feature importance is expressed as the mean percentage increase in node purity (%IncMSE). The variance explained (R^2^) by the random forest model was calculated using out-of-bag predictions.

Potential confounders considered for inclusion in the models were age, obesity (yes or no), smoking status (yes or no), mother’s menarche age, mother’s delivery age, and mode of delivery (vaginal or cesarean).

To account for multiple comparisons due to testing 12 PFASs across three outcome variables (T3, T4, rT3) and three groups, we applied the Benjamini–Hochberg false discovery rate (FDR) correction with a threshold of Q < 0.20. Associations with FDR-adjusted Q values < 0.20 were considered statistically significant after correction.

The analysis utilized IBM SPSS (version 26, Armonk, NY, USA), considering P < 0.05 as significant.

## 3 Results

As shown in Table 1, PFHpA, PFOA, PFNA, PFDA, PFUnDA, PFDoDA, PFBS, PFHxS, PFHpS, PFOS, 6:2Cl-PFESA, and 8:2Cl- PFESA were the main PFASs, whose detection rate was above 95%. Among the 25 PFASs, PFBA, PFPeA, PFHxA, HFPO-DA, PFPeS (4:2FTS) 6:2FTS, 8:2FTS, NaDONA, PFEESA, 6:2 diPAP, 8:2 diPA), and fluorotelomer phosphate diester (6:2/8:2 diPAP) were detected in less than 60% of the samples in all groups, which were excluded from further studies. In all the PFASs, the highest level of PFASs was PFOA (mean: 15.30 ng/mL, 15.00 ng/mL and 17.96 ng/mL in the control, CPP and IPP groups, respectively), followed by PFOS (mean: 4.34 ng/mL, 4.91 ng/mL and 5.48 ng/mL in the control, CPP and IPP groups, respectively). The median values of PFBA, PFPeA, PFHxA, HFPO-DA, PFPeS, 4:2FTS, 6:2FTS, 8:2FTS, PFEESA, 6:2 diPAP, 8:2diPAP and 6:2/8:2 diPAP were below the LOQ.

**TABLE 1:** Descriptive statistics of PFASs in control, CPP and IPP groups.

	Controls (n = 401)			Cases CPP (n = 226)			Cases IPP (n = 316)		
PFASs (ng/mL)	DR (%)	Median	25th–75th percentile	DR (%)	Median	25th–75th percentile	DR (%)	Median	25th–75th percentile
PFBA	38.40	<LOQ	<LOQ -0.13	30.97	<LOQ	<LOQ-0.11	45.25	<LOQ	<LOQ-0.15
PFPeA	0.01	< LOQ	<LOQ- < LOQ	2.65	<LOQ	<LOQ- < LOQ	6.01	<LOQ	<LOQ- < LOQ
PFHxA	48.38	<LOQ	<LOQ -0.26	42.48	<LOQ	<LOQ-0.25	44.30	<LOQ	<LOQ-0.24
PFHpA	100.00	0.14	0.09–0.22	100.00	0.13	0.09–0.19	100.00	0.15	0.10–0.22
PFOA	100.00	15.30	11.07–20.89	100.00	15.00	12.05–19.55	100.00	17.96	12.77–24.13
PFNA	100.00	1.63	1.12–2.50	100.00	1.61	1.10–2.25	100.00	1.83	1.23–3.23
PFDA	100.00	0.91	0.62–1.55	100.00	1.02	0.59–1.58	100.00	1.15	0.67–2.16
PFUnDA	100.00	0.72	0.45–1.17	100.00	0.73	0.46–1.15	100.00	0.85	0.49–1.48
PFDoDA	99.00	0.07	0.04–0.11	98.67	0.06	0.04–0.11	100.00	0.08	0.04–0.15
HFPO-DA	1.00	< LOQ	<LOQ- < LOQ	0.44	<LOQ	<LOQ- < LOQ	0.63	<LOQ	<LOQ- < LOQ
PFBS	98.00	0.10	0.07–0.15	100.00	0.13	0.08–0.19	100.00	0.14	0.10–0.21
PFPeS	2.49	< LOQ	<LOQ- < LOQ	1.33	<LOQ	<LOQ- < LOQ	1.90	<LOQ	<LOQ- < LOQ
PFHxS	100.00	2.80	1.83–3.95	100.00	3.02	2.16–3.93	100.00	3.33	2.41–4.86
PFHpS	96.26	0.16	0.11–0.23	97.35	0.16	0.11–0.22	100.00	0.18	0.13–0.27
PFOS	98.25	4.34	2.62–6.94	96.02	4.91	2.92–7.41	96.52	5.48	3.32–10.33
4:2FTS	0.00	< LOQ	<LOQ- < LOQ	0.88	<LOQ	<LOQ- < LOQ	2.53	<LOQ	<LOQ- < LOQ
6:2FTS	8.48	< LOQ	<LOQ- < LOQ	7.08	<LOQ	<LOQ- < LOQ	19.94	<LOQ	<LOQ- < LOQ
8:2FTS	0.75	< LOQ	<LOQ- < LOQ	1.33	<LOQ	<LOQ- < LOQ	1.27	<LOQ	<LOQ- < LOQ
NaDONA	0.25	< LOQ	<LOQ- < LOQ	0.00	<LOQ	<LOQ- < LOQ	0.32	<LOQ	<LOQ- < LOQ
6:2Cl-PFESA	99.75	1.47	0.92–2.62	100.00	2.01	1.12–3.50	100.00	2.34	1.48–4.70
8:2Cl- PFESA	100.00	0.02	0.01–0.04	100.00	0.03	0.02–0.05	100.00	0.03	0.02–0.08
PFEESA	0.00	<LOQ	<LOQ- < LOQ	0.00	<LOQ	<LOQ- < LOQ	0.32	<LOQ	<LOQ- < LOQ
6:2 diPAP	0.25	<LOQ	<LOQ- < LOQ	0.44	<LOQ	<LOQ- < LOQ	0.95	<LOQ	<LOQ- < LOQ
8:2 diPAP	0.00	<LOQ	<LOQ- < LOQ	0.44	<LOQ	<LOQ- < LOQ	0.63	<LOQ	<LOQ- < LOQ
6:2/8:2 diPAP	0.00	<LOQ	<LOQ- < LOQ	0.00	<LOQ	<LOQ- < LOQ	0.95	<LOQ	<LOQ- < LOQ

Abbreviation: PFASs, per- and polyfluoroalkyl substances; CPP, central precocious puberty; IPP, incomplete precocious puberty; LOQ, limit of quantification.

We summarized the characteristics of the girls in the three groups and compared THs levels correlating with the studied characteristics, such as age, obesity or not, either parent’s smoking, mothers’ menarche age and delivery age (Table 2). The T3, T4, and rT3 levels of all the participants were 2.20 (1.63, 2.89) ng/mL, 213.62 (159.57, 316.00) ng/mL, and 0.34 (0.13, 0.41) ng/mL, respectively. T3 values were similar across the body mass index (BMI), mother’s menarche and delivery age. T4 values were similar across BMI, and either parent’s smoking status. rT3 values were similar across BMI, either parent’s smoking status and mother’s menarche age. Therefore, we subsequently performed logistic and multiple linear regression analyses, adjusting for factors such as age, BMI, parent’s smoking, mother’s menarche age, and mother’s delivery age. T3 values decreased significantly with increasing age, and T4 showed the opposite trend. T4 concentrations were lower in mothers with a younger age at menarche and older age at delivery. The levels of rT3 correlated with the studied characteristics of mother’s delivery age. Girls whose mothers gave birth to them at older age had a lower rT3.

**TABLE 2:** Correlations between demographic characteristics and THs concentrations in serum of all the participants.

Characteristics	No.	100%	Measures of THs		
			T3 (ng/mL)	T4 (ng/mL)	rT3 (ng/mL)
TH levels, mean (SD)	943	100	2.20 (1.63, 2.89)	213.62 (159.57, 316.00)	0.34 (0.13, 0.41)
Age, y					
7–8	229	24.3	2.42 (1.74, 3.32)**	185.66 (148.28, 260.16)***	0.35 (0.18, 0.52)***
8–9	578	61.3	2.18 (1.65, 2.83)	214.74 (164.55, 309.01)	0.32 (0.09, 0.38)
9–10	136	14.4	2.01 (1.58, 2.55)	291.86 (198.74, 391.61)	0.35 (0.29, 0.35)
Obesity					
Yes	93	9.9	2.33 (1.83, 3.03)	201.00 (148.65, 285.23)	0.33 (0.08, 0.45)
No	850	90.1	2.18 (1.62, 2.85)	214.11 (162.07, 317.96)	0.34 (0.13, 0.41)
Smoking of parent					
Yes	472	50.1	2.11 (1.57, 2.85)*	221.71 (158.34, 335.29)	0.33 (0.12, 0.37)
No	471	49.9	2.25 (1.72, 2.92)	208.87 (164.04, 292.41)	0.35 (0.13, 0.46)
Mother’s menarche age, y					
<13	101	10.7	2.15 (1.75, 2.97)	180.66 (136.81, 231.05)***	0.28 (0.07, 0.43)
≥13	842	89.3	2.21 (1.62, 2.87)	218.18 (164.25, 322.28)	0.35 (0.13, 0.41)
Mother’s delivery age, y					
≥30	127	13.5	2.38 (1.75, 3.04)	201.00 (153.54, 254.00)*	0.26 (0.07, 0.38)*
<30	816	86.5	2.17 (1.63, 2.84)	215.68 (162.10, 322.63)	0.35 (0.13, 0.41)

Abbreviation: THs, thyroid hormones; T3, 3,5,3′-triiodothyronine; T4, thyroxin; rT3, 3,3′,5′-triiodothyronine (rT3).

ns, p ≥ 0.05; *, p < 0.05; **, p < 0.01; ***, p < 0.001.

**FIGURE 1:**
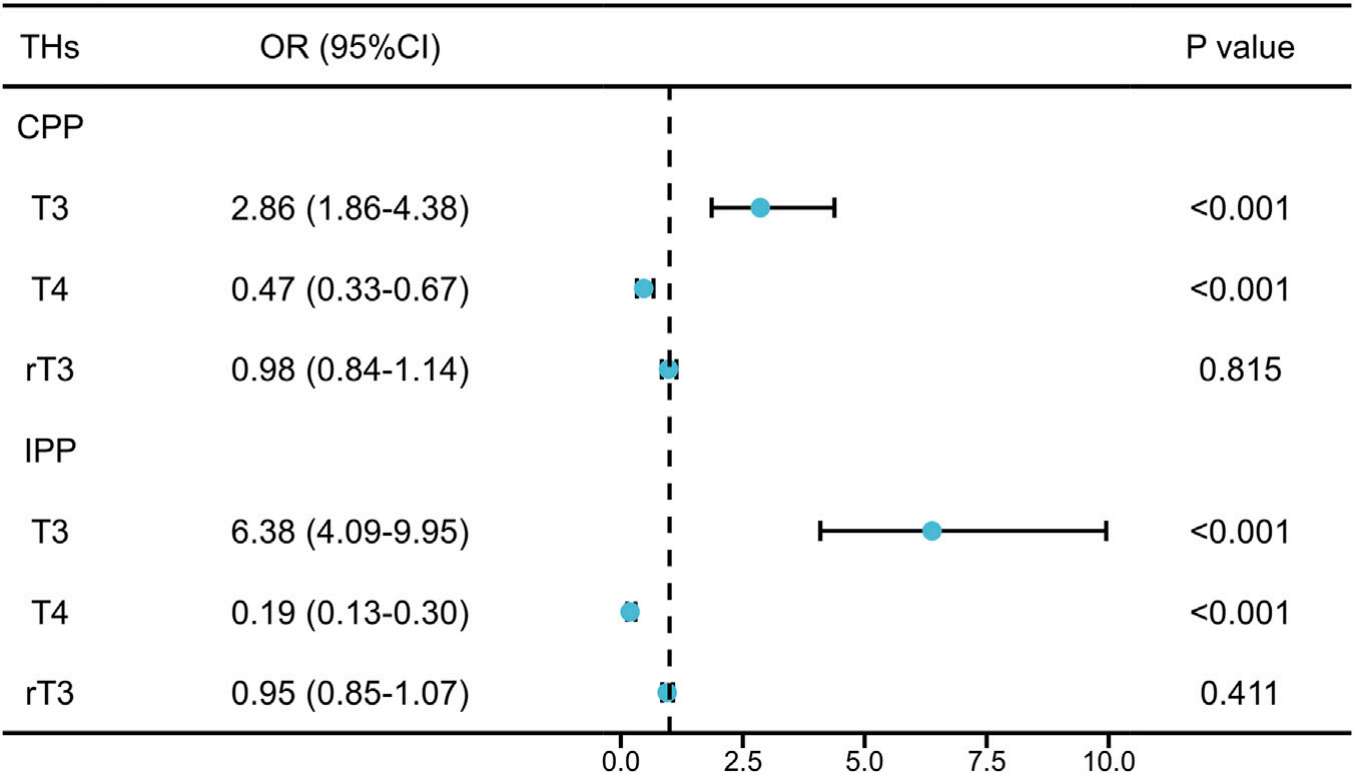
Association Between THs Values and Odds of CPP/IPP. Abbreviation: THs, thyroid hormones; CPP, central precocious puberty; IPP, incomplete precocious puberty; T3, 3,5,3′-triiodothyronine; T4, thyroxin; rT3, 3,3′,5′-triiodothyronine (rT3); OR, odds ratios. ns, p ≥ 0.05; *, p < 0.05; **, p < 0.01; ***, p < 0.001 Models adjusted for age, parent smoking, mother’s menarche age and mother’s delivery age. All thyroid hormone concentrations were transformed using the natural logarithm.

The impact of THs on CPP and IPP was evaluated using logistic regression (Figure 1). After being adjusted for age, BMI, parent’s smoking, mother’s menarche age, and mother’s delivery age (Li et al., 2018), notable connections were identified in T3 (OR: 2.86, 95% CI: 1.86–4.38, and OR: 6.38, 95%CI: 4.09–9.95) with CPP and with IPP. While adverse associations were observed in T4 (OR: 0.47, 95%CI: 0.33–0.67, and OR: 0.19, 95%CI: 0.13–0.30) with CPP and with IPP. However, rT3 had no significant associations with CPP and IPP. These results indicated that higher T3 levels were associated with increased odds of CPP and IPP, with a stronger association observed for IPP. Nevertheless, the opposite conclusion was shown in T4.

**FIGURE 2:**
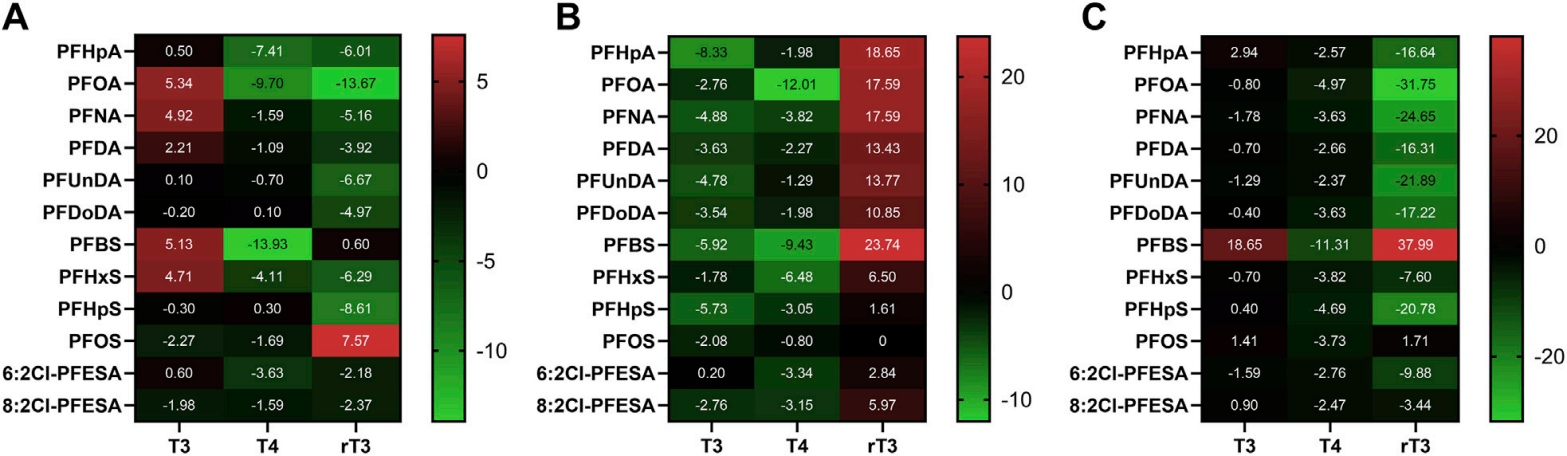
Associations Between Various PFASs and TH Concentrations. **(A)** Associations Between Various PFASs and TH Concentrations in control group. **(B)** Associations Between Various PFASs and TH Concentrations in CPP group. **(C)** Associations Between Various PFASs and TH Concentrations in IPP group. Abbreviation: THs, thyroid hormones; CPP, central precocious puberty; IPP, incomplete precocious puberty; T3, 3,5,3′-triiodothyronine; T4, thyroxin; rT3, 3,3′,5′-triiodothyronine (rT3); OR, odds ratios.

On account of the differences observed, cases and control subjects were analyzed of the associations between PFASs and THs in respective groups. We put the 12 PFASs of detection rate of >60% into the multiple linear regression model, and adjusted for age, BMI, parent’s smoking, mother’s menarche age, and mother’s delivery age. As shown in Table 3 and Figure 2A, in the control group, T4 levels were significantly inversely associated with PFHpA (%change = −7.41; 95% CI, −14.27 to 0.00; *P* = 0.049), PFOA (%change = −9.70; 95% CI, −17.39 to −1.19; *P* = 0.026), and PFBS (%change = −13.93; 95% CI, −19.67 to −7.78; *P* = 0.000). In CPP group, T3 concentrations had negative tendencies toward significance for PFHpA (%change = −8.33; 95% CI, −14.96 to −1.29; *P* = 0.022). While T4 had negative tendencies to significance for PFOA (%change = −12.01; 95% CI, −21.10 to −1.88; *P* = 0.022), PFBS (%change = −9.43; 95% CI, −16.39 to −1.88; *P* = 0.016) and PFHxS (%change = −6.48; 95% CI, −11.84 to −0.70; *P* = 0.029) (Table 4 and Figure 2B). In IPP group, PFBS had positive tendencies toward significance with T3 (%change = 18.65; 95% CI, 9.42 to 28.66; *P* = 0.000), however had negative tendencies toward significance with T4 (%change = −11.31; 95% CI, −16.64 to −5.64; *P* = 0.000). In addition, T4 was negatively associated with PFOS (%change = −3.73; 95% CI, −6.01 to −1.29; *P* = 0.002). rT3 concentrations had negative tendencies toward significance with several PFASs only in IPP group, such as PFOA levels (%change = -31.75; 95% CI, −53.14 to −0.50; *P* = 0.047), PFNA (%change = −24.65; 95% CI, −42.42 to −1.39; *P* = 0.040) and PFUnDA (%change = −21.89; 95% CI, −37.31 to −2.66; *P* = 0.028) (Table 5 and Figure 2C). Multiple comparisons (12 PFASs × 3 thyroid hormones × 3 groups) increase the risk of Type I error. To address this, we applied Benjamini-Hochberg FDR correction with a threshold of P < 0.20. The raw P-values and FDR-adjusted Q-values for all 108 comparisons were shown in Supplementary Tables 1–3. All significant associations reported remained consistent after FDR correction.

**TABLE 3:** Associations Between Various PFASs and TH Concentrations in control group.

Analytes	T3		T4		rT3	
	%change (95%CI)	*P* value	%change (95%CI)	*P* value	% change (95% CI)	*P* value
PFHpA	0.50 (−7.23, 8.87)	0.906	−7.41 (−14.27, 0.00)	0.049*	−6.01 (−21.10, 11.96)	0.487
PFOA	5.34 (−4.11, 15.72)	0.279	−9.70 (−17.39, −1.19)	0.026*	−13.67 (−29.67, 5.87)	0.158
PFNA	4.92 (−2.96, 13.31)	0.228	−1.59 (−8.70, 6.08)	0.673	−5.16 (−19.99, 12.41)	0.541
PFDA	2.12 (−4.30, 8.98)	0.530	−1.09 (−7.04, 5.34)	0.736	−3.92 (−16.64, 10.85)	0.584
PFUnDA	0.10 (−6.11, 6.72)	0.975	−0.70 (−6.57, 5.55)	0.826	−6.67 (−18.70, 7.14)	0.327
PFDoDA	−0.20 (−6.48, 6.50)	0.952	0.10 (−6.01, 6.50)	0.978	−4.97 (−17.55, 9.53)	0.481
PFBS	5.13 (−2.27, 13.20)	0.179	−13.93 (−19.67, −7.78)	0.000***	0.60 (−14.27, 18.18)	0.939
PFHxS	4.71 (−1.69, 11.52)	0.149	−4.11 (−9.70, 1.82)	0.174	−6.29 (−18.29, 7.47)	0.351
PFHpS	−0.30 (−8.52, 8.55)	0.936	0.30 (−7.60, 8.87)	0.936	−8.61 (−24.12, 10.08)	0.343
PFOS	−2.27 (−6.67, 2.22)	0.318	−1.69 (−5.92, 2.74)	0.444	7.57 (−2.57, 18.89)	0.146
6:2Cl-PFESA	0.60 (−4.40, 5.87)	0.820	−3.63 (−8.24, 1.31)	0.144	−2.18 (−12.54, 9.42)	0.695
8:2Cl-PFESA	−1.98 (−7.04, 3.25)	0.446	−1.59 (−6.39, 3.46)	0.534	−2.37 (−12.89, 9.42)	0.681

Abbreviation: THs, thyroid hormones; T3, 3,5,3′-triiodothyronine; T4, thyroxin; rT3, 3,3′,5′-triiodothyronine (rT3).

Models adjusted for age, parent smoking, mother’s menarche age and mother’s delivery age.

Abbreviation: THs, thyroid hormones; T3, 3,5,3′-triiodothyronine; T4, thyroxin; rT3, 3,3′,5′-triiodothyronine (rT3).

Models adjusted for age, parent smoking, mother’s menarche age and mother's delivery age.

*p < 0.05; ***p < 0.001.

**TABLE 4:** Associations Between Various PFASs and TH Concentrations in CPP group.

Analytes	T3		T4		rT3	
	%change (95%CI)	*P*	% change (95% CI)	*P*	% change (95% CI)	*P*
PFHpA	−8.33 (−14.96, −1.29)	0.022^*^	−1.98 (−8.61, 5.13)	0.573	18.65 (−5.26, 48.59)	0.136
PFOA	−2.76 (−13.58, 9.42)	0.641	−12.01 (−21.10, −1.88)	0.022*	17.59 (−17.39, 67.53)	0.367
PFNA	−4.88 (−11.84, 2.63)	0.200	−3.82 (−10.42, 3.25)	0.282	17.59 (−6.39, 47.70)	0.162
PFDA	−3.63 (−9.15, 2.33)	0.225	−2.27 (−7.50, 3.25)	0.413	13.43 (−5.07, 35.53)	0.165
PFUnDA	−4.78 (−10.42, 1.21)	0.115	−1.29 (−6.76, 4.50)	0.652	13.77 (−5.26, 36.62)	0.167
PFDoDA	−3.54 (−9.15, 2.43)	0.243	−1.98 (−7.32, 3.67)	0.491	10.85 (−7.41, 32.71)	0.260
PFBS	−5.92 (−13.76, 2.53)	0.164	−9.43 (−16.39, −1.88)	0.016*	23.74 (−4.59, 60.32)	0.108
PFHxS	−1.78 (−7.96, 4.81)	0.578	−6.48 (−11.84, −0.70)	0.029*	6.50 (−12.28, 29.43)	0.521
PFHpS	−5.73 (−13.06, 2.22)	0.155	−3.05 (−10.15, 4.60)	0.428	1.61 (−20.55, 29.82)	0.901
PFOS	−2.08 (−5.35, 1.31)	0.227	−0.80 (−3.92, 2.43)	0.610	0.00 (−9.70, 10.85)	0.996
6:2Cl-PFESA	0.20 (−5.35, 5.97)	0.952	−3.34 (−8.33, 1.82)	0.196	2.84 (−13.15, 21.77)	0.744
8:2Cl-PFESA	−2.76 (−7.32, 2.02)	0.248	−3.15 (−7.41, 1.21)	0.156	5.97 (−8.24, 22.38)	0.430

Abbreviation: THs, thyroid hormones; T3, 3,5,3′-triiodothyronine; T4, thyroxin; rT3, 3,3′,5′-triiodothyronine (rT3).

Models adjusted for age, parent smoking, mother’s menarche age and mother’s delivery age.

*p < 0.05.

**TABLE 5:** Associations Between Various PFASs and TH Concentrations in IPP group.

Analytes	T3		T4		rT3	
	%change (95%CI)	*P*	% change (95% CI)	*P*	% change (95% CI)	*P*
PFHpA	2.94 (−3.63, 10.08)	0.386	−2.57 (−7.41, 2.53)	0.317	−16.64 (−36.05, 8.55)	0.176
PFOA	−0.80 (−9.79, 9.09)	0.867	−4.97 (−11.66, 2.22)	0.168	−31.75 (−53.14, −0.50)	0.047^*^
PFNA	−1.78 (−8.24, 5.13)	0.602	−3.63 (−8.52, 1.51)	0.165	−24.65 (−42.42, −1.39)	0.040^*^
PFDA	−0.70 (−5.73, 4.50)	0.780	−2.66 (−6.39, 1.31)	0.188	−16.31 (−31.89, 2.84)	0.089
PFUnDA	−1.29 (−6.57, 4.39)	0.654	−2.37 (−6.48, 1.82)	0.261	−21.89 (−37.31, −2.66)	0.028^*^
PFDoDA	−0.40 (−5.35, 4.92)	0.889	−3.63 (−7.32, 0.30)	0.069	−17.22 (−32.56, 1.61)	0.071
PFBS	18.65 (9.42, 28.66)	0.000***	−11.31 (−16.64, −5.64)	0.000***	37.99 (−0.80, 91.75)	0.056
PFHxS	−0.70 (−6.95, 5.97)	0.834	−3.82 (−8.52, 1.11)	0.127	−7.60 (−28.82, 19.96)	0.551
PFHpS	0.40 (−7.04, 8.55)	0.912	−4.69 (−10.15, 1.11)	0.112	−20.78 (−41.78, 7.68)	0.137
PFOS	1.41 (−1.78, 4.71)	0.378	−3.73 (−6.01, −1.29)	0.002^**^	1.71 (−10.42, 15.49)	0.792
6:2Cl-PFESA	−1.59 (−6.48, 3.67)	0.552	−2.76 (−6.48, 1.21)	0.170	−9.88 (−26.66, 10.85)	0.324
8:2Cl-PFESA	0.90 (−3.15, 5.02)	0.676	−2.47 (−5.45, 0.60)	0.113	−3.44 (−17.96, 13.54)	0.669

Abbreviation: THs, thyroid hormones; T3, 3,5,3′-triiodothyronine; T4, thyroxin; rT3, 3,3′,5′-triiodothyronine (rT3).

Models adjusted for age, parent smoking, mother’s menarche age and mother’s delivery age.

*p < 0.05, **p < 0.01, ***p < 0.001.

In this equation, we picked certain PFASs closely related to THs and analyzed their relationship with THs at different concentrations. Table 6 showed the association between quartiles of PFOA and PFBS and THs, where the reference is the lowest quartile of exposure. The concentration range of the highest quartile of 19.56–85.03 ng/mL of PFOA was associated with a 6.57% decrease in T4 in the control group (95% CI, −11.22 to −1.59; *P* = 0.010), and 4.50% decrease in T4 in the CPP group (95% CI, −8.61 to −0.20; *P* = 0.041), but the association showed limitation in the IPP group. Associations between PFBS and THs were found mainly in control and IPP groups. Interquartile contrasts of 0.20–0.58 ng/mL in PFBS were respectively associated with a 9.70% drop in T4 (95% CI, −13.41 to −5.82, *P* = 0.000) in the control group and 6.29% drop in T4 (95%CI, −9.34 to −3.25, *P* = 0.000) in the IPP group. The positive association was obvious in PFBS with T3 in 0.14–0.58 ng/mL in the IPP group, while in other groups, the associations were not evident (Table 6).

**TABLE 6:** Association between selected PFASs concentration quartiles and THs levels in control, CPP and IPP groups respectively.

Analytes	T3		T4		rT3	
	% change (95% CI)	*P*	% change (95% CI)	*P*	% change (95% CI)	*P*
Control						
PFOA						
1	Reference		Reference		Reference	
2	−10.51 (−21.89, 2.53)	0.110	1.31 (−10.24, 14.34)	0.832	−4.21 (−29.11, 29.30)	0.775
3	3.98 (−2.18, 10.52)	0.213	−2.37 (−7.96, 3.56)	0.416	−5.64 (−19.18, 10.30)	0.467
4	1.11 (−4.21, 6.82)	0.676	−6.57 (−11.22, −1.59)	0.010*	−1.98 (−11.93, 9.09)	0.712
PFBS						
1	Reference		Reference		Reference	
2	6.40 (−7.41, 22.14)	0.381	−15.80 (−25.77, −4.50)	0.008^**^	45.35 (5.55, 99.97)	0.022^*^
3	1.31 (−4.69, 7.57)	0.680	−4.30 (−9.70, 1.51)	0.142	−5.54 (−18.94, 9.97)	0.460
4	2.33 (−2.37, 7.25)	0.342	−9.70 (−13.41, −5.82)	0.000^***^	−2.84 (−6.95, 13.77)	0.576
CPP						
PFOA						
1	Reference		Reference		Reference	
2	−8.97 (−21.10, 5.13)	0.198	−3.25 (−14.62, 9.75)	0.606	−6.29 (−39.95, 46.37)	0.773
3	−1.09 (−7.78, 6.08)	0.759	−5.26 (−11.31, 1.11)	0.102	4.50 (−16.81, 31.13)	0.705
4	−2.08 (−7.13, 3.15)	0.424	−4.50 (−8.61, −0.20)	0.041^*^	−2.47 (−15.63, 12.64)	0.730
PFBS						
1	Reference		Reference		Reference	
2	−8.70 (−21.42, 5.97)	0.230	−2.66 (−13.50, 9.53)	0.652	5.02 (−32.36, 62.91)	0.826
3	−23.05 (−39.41, −2.27)	0.032^*^	7.90 (−13.24, 34.18)	0.491	8.33 (−45.28, 114.68)	0.817
4	−0.10 (−5.54, 5.76)	0.981	−4.40 (−8.79, 0.20)	0.063	9.31 (−6.76, 28.15)	0.270
IPP						
PFOA						
1	Reference		Reference		Reference	
2	−1.59 (−14.44, 13.20)	0.821	−4.69 (−13.32, 4.92)	0.326	−38.25 (−61.63, −0.60)	0.047^*^
3	1.11 (−4.88, 7.57)	0.713	−0.50 (−5.35, 4.50)	0.832	−14.53 (−33.83, 10.41)	0.228
4	−0.70 (−4.59, 3.25)	0.712	−2.57 (−5.64, 0.70)	0.125	−18.70 (−30.93, −4.30)	0.013^*^
PFBS						
1	Reference		Reference		Reference	
2	−0.90 (−13.06, 13.09)	0.895	−4.50 (−13.06, 4.92)	0.333	−0.70 (−44.35, 77.18)	0.980
3	10.41 (4.60, 16.53)	0.000***	−5.73 (−10.33, −1.00)	0.019*	5.23 (−18.70, 36.21)	0.697
4	8.00 (4.39, 11.74)	0.000***	−6.29 (−9.34, −3.25)	0.000***	13.31 (−4.59, 34.58)	0.152

Abbreviation: THs, thyroid hormones; T3, 3,5,3′-triiodothyronine; T4, thyroxin; rT3, 3,3′,5′-triiodothyronine (rT3).

Models adjusted for age, parent smoking, mother’s menarche age and mother’s delivery age.

ns, p ≥ 0.05; *, p < 0.05; **, p < 0.01; ***, p < 0.001.

To evaluate potential effect modification, we conducted interaction analyses by adding product terms (PFAS × obesity status and PFAS × maternal age at menarche) to the multiple linear regression models. We examined whether the associations between PFASs and THs were modified by obesity status or maternal age at menarche. Stratified analyses were performed by obesity status (yes vs. no) and by maternal age at menarche (<13 years vs. ≥13 years) to examine whether PFAS–TH associations differed across these subgroups.

Interaction terms for PFAS × obesity and PFAS × maternal age at menarche were not statistically significant for any PFAS–TH pair (all interaction P > 0.05), indicating no significant effect modification. Stratified analyses by obesity status yielded similar point estimates and confidence intervals between obese and non-obese girls, although the sample size in the obese subgroup (n = 93) limited statistical power. Similarly, stratification by maternal age at menarche (<13 years vs. ≥13 years) did not reveal consistent evidence of interaction. These results suggest that the observed PFAS–TH associations were not significantly modified by obesity or maternal age at menarche in our study population.

**FIGURE 3:**
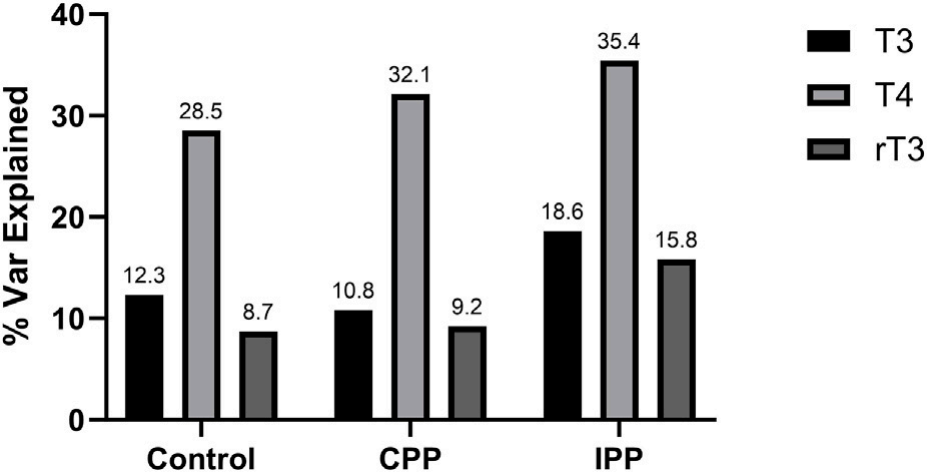
Analysis of the predictive ability of the random forest model for PFASs under different TH conditions Abbreviation: THs, thyroid hormones; CPP, central precocious puberty; IPP, incomplete precocious puberty; T3, 3,5,3′-triiodothyronine; T4, thyroxin; rT3, 3,3′,5′-triiodothyronine (rT3); OR, odds ratios.

**FIGURE 4:**
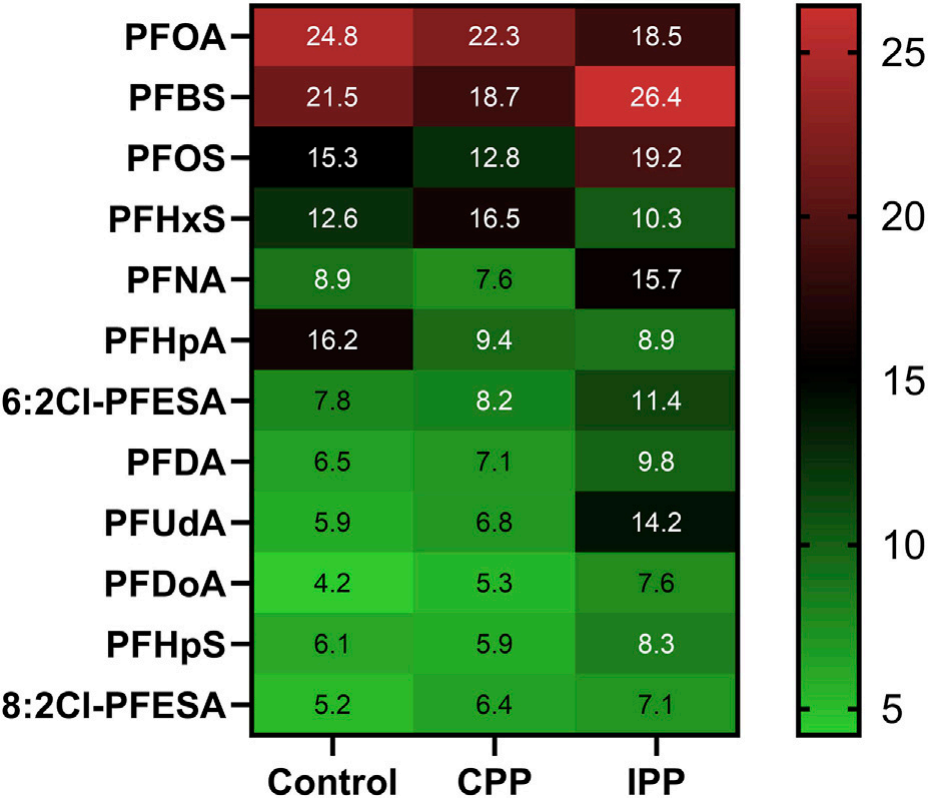
Characteristic importance of each PFASs for T4 prediction Abbreviation: THs, thyroid hormones; CPP, central precocious puberty; IPP, incomplete precocious puberty; T3, 3,5,3′-triiodothyronine; T4, thyroxin; rT3, 3,3’,5’-triiodothyronine (rT3); OR, odds ratios.

VIF values for all 12 PFASs were below 5, indicating acceptable levels of multicollinearity. Random forest analysis revealed that PFASs had the strongest predictive power for T4, explaining 28.5%, 32.1%, and 35.4% of T4 variance in the control, CPP, and IPP groups, respectively. The predictive power for T3 and rT3 was relatively weaker (explaining 8.7%–18.6% of variance) (Figure 3). Notably, the predictive power of PFASs for all three THs was higher in the IPP group than in the other two groups, suggesting that IPP girls may show greater susceptibility to PFAS-associated thyroid hormone alterations. Subsequently, we evaluated the feature importance (as a percentage, %IncMSE) of each PFAS in predicting T4. Random forest feature importance analysis showed that PFOA and PFBS were the two PFASs with the greatest impact on THs across all groups, with mean %IncMSE of 21.9% and 22.2%, respectively (Table 7 and Figure 4). This outcome is closely aligned with the results from multiple linear regression. The conditional permutation importance analysis yielded a ranking of feature importance consistent with the %IncMSE results, with PFOA and PFBS remaining the top two contributors to T4 prediction across all groups.

**TABLE 7:** Characteristic importance of each PFAS for T4 prediction.

PFASs	%IncMSE			
	Control	CPP	IPP	Mean
PFOA	24.8	22.3	18.5	21.9
PFBS	21.5	18.7	26.4	22.2
PFOS	15.3	12.8	19.2	15.8
PFHxS	12.6	16.5	10.3	13.1
PFNA	8.9	7.6	15.7	10.7
PFHpA	16.2	9.4	8.9	11.5
6:2Cl-PFESA	7.8	8.2	11.4	9.1
PFDA	6.5	7.1	9.8	7.8
PFUdA	5.9	6.8	14.2	9
PFDoA	4.2	5.3	7.6	5.7
PFHpS	6.1	5.9	8.3	6.8
8:2Cl-PFESA	5.2	6.4	7.1	6.2

## 4 Discussion

Few studies have investigated the associations among PFASs, CPP, IPP and thyroid function. Based on our literature review, this research is among the initial studies to investigate the link between thyroid function and PFASs in girls with CPP or IPP. We examined the connections between PFASs and THs in the CPP, IPP, and control groups.

PFASs are synthetic organic compounds characterized by carbon chains that are either polyfluorinated or perfluorinated, along with various functional groups (Buck et al., 2011). Owing to their hydrophobic and oleophobic properties, PFASs have been used in manufacturing and daily applications (Vecitis et al., 2010). A large volume of PFASs is produced and consumed in China, which might cause persistence, bioaccumulation, and probable poisonousness, negatively affecting human health and raising concerns regarding public health. The atmospheric environment and wastewater are notable exposure pathways through which PFASs impact humans and are the main compartments for PFASs to be transported and transformed (Wang et al., 2020; Lu et al., 2021). In our study, the highest concentrations of PFASs were PFOA, followed by PFOS. By contrast, in another study, PFBA, PFHxA, PFHpA, PFOA, and PFOS were the most common PFASs in wastewater (Lu et al., 2021). Still another study indicated that PFPeA, PFOA, and PFBA were important sources, acting as the primary PFAS in China (Wang et al., 2020). We observed that the median concentration of PFBA (<LOQ) in the three groups was lower than that in Beijing (0.1 ng/mL) (Li et al., 2023) and Guangdong (0.7 ng/mL) (Cai et al., 2020), China. The median PFBS concentration in our control group (0.10 ng/mL) fell between levels reported for the and Beijing (0.14 ng/mL) (Li et al., 2023) and Shanghai Birth Cohort (0.03 ng/mL) (Nian et al., 2020). PFOS levels (4.34 ng/mL) aligned closely with Beijing data (4.09 ng/mL) (Li et al., 2023), yet remained substantially below concentrations observed in Massachusetts, US (24.0 ng/mL) (Preston et al., 2018) and Spain (6.05 ng/mL) (Mamsen et al., 2017), while exceeding those from Colorado, US (2.4 ng/mL) (Starling et al., 2017). For 6:2 Cl-PFESA, median values of 0.02 ng/mL (controls), 0.03 ng/mL (CPP), and 0.03 ng/mL (IPP) were markedly below estimates from both Beijing (0.09 ng/mL) (Li et al., 2023) and in Wuhan (1.89 ng/mL) (Pan et al., 2017). These different consequences may be due to various physical characteristics, industrial degrees, and exposure situations across species and areas.

We observed differences in THs levels in the CPP, IPP and control groups. Several studies have reported differences in thyroid function. Evidence indicates that the levels of serum free thyroxine (fT4) are notably lower in groups with CPP than in those without. Studies have shown that hyperthyrotropinemia is more common in the CPP group compared to the non-CPP group, and there is an independent positive correlation between peak LH levels and serum TSH concentration (Jung et al., 2019). Additional studies have identified that TSH and T3 levels are increased in obese children when compared to control groups, but T4 levels show no significant difference (Shalitin et al., 2009). This phenomenon probably reflects the influence of obesity on THs levels (Bastemir et al., 2007). In individuals with obesity, changes in thyroid hormones can arise from various causes like autoimmunity, TSH receptor gene mutations, leptin, resistance to thyroid hormones, and issues with mitochondrial function (Pacifico et al., 2012).

We analyzed the heterogeneity of these characteristics for T3, T4, and rT3. Characteristics including age, parent’s tobacco smoking, mother’s menarche age, and mother’s delivery age affected the aforementioned THs levels. As a result, these variables were considered confounders in the statistical analyses. According to the literature, factors like gestational age, maternal smoking, and birth weight have been linked to THs (Li et al., 2018). Other researchers have also found associations between THs and delivery mode (Aktas et al., 2017), alcohol consumption (Darnerud et al., 2010), and maternal BMI (Collares et al., 2017).

T3 increased the risk of CPP and IPP, and the risk was similar after adjustment in this study (for CPP: OR = 2.86, 95% CI = 1.86–4.38; for IPP: OR = 6.38, 95% CI = 4.09–9.95). T4 had a low inverse risk in both CPP and IPP (for CPP: OR = 0.47, 95% CI = 0.33–0.67; for IPP: OR = 0.19, 95% CI = 0.13–0.30). LH and TSH share a common glycoprotein architecture—consisting of a universal α subunit coupled with unique β subunits—which provides a structural rationale for the cross-talk between reproductive and thyroid axes (Vassart et al., 2004). Due to this similarity, LH can strongly attach to the TSH receptor. TSH is also capable of binding to LH or FSH receptors, which can result in ovarian hyperstimulation and trigger early puberty (Vassart et al., 2004). Another mechanism underlying the association between thyroid function and sexual development is related to obesity. Adiposity represents a well-established accelerator of pubertal onset (Ahmed et al., 2009), with established links to both CPP and elevated circulating TSH (Cho et al., 2018). The adipokine leptin further serves as a critical modulator of thyroid homeostasis (Zimmermann-Belsing et al., 2003), influencing hypothalamic TRH release while enhancing peripheral T3 generation via stimulation of T4-to-T3 conversion (Cusin et al., 2000). Leptin attaches to its receptor in hypothalamic neurons that generate GnRH, which is then transported to the pituitary via the portal vascular system, prompting the release of LH and FSH. Gonadotropins stimulate the production of sex steroid hormones and mark the onset of puberty (Pankov, 1999).

Several studies have identified strong links between exposure to PFASs and negative immune effects in children (Sunderland et al., 2019). A recent comprehensive review of literature on children’s health found positive links between PFAS exposure and dyslipidemia, immune function, kidney health, and the age of onset of menstruation (Rappazzo et al., 2017). There were discrepancies in the associations between different PFASs and THs in CPP, IPP and healthy groups. Similarly, the results were not consistently described by all studies, and extreme inconsistency was observed when a comparison between long and short PFASs was considered. Therefore, the characteristics of subjects, including exposed areas, occupations, age, sex, and population, should be considered in this study. Our results showed an inverse association between PFOA and T4 levels in healthy and CPP girls, and the THs have no significance with PFOA in the IPP cohort. Similar to another study, Shrestha et al. reported that an increase in serum PFOA was associated with an increase in serum total T3 in women (Shrestha et al., 2015). In the multiple linear regression, after adjustment, a negative association between PFBS and T4 was observed in all the groups, while in IPP group, there is positive significance between PFBS and T3. Evidence indicates that PFBS was positively associated with maternal TSH levels during the second trimester, a pattern distinct from most other PFASs, which showed negative associations with TSH and fT4 in a 2025 prospective birth cohort study in Wuxi, China (Zhou et al., 2025). This suggests that PFBS may be uniquely associated with stimulation of the hypothalamic–pituitary–thyroid axis, potentially leading to subclinical hypothyroidism. Recent mechanistic evidence further supports the thyroid-disrupting potential of PFBS. Zhou et al. (2025) demonstrated that PFBS exhibits significant binding interaction with thyroid hormone-related proteins, and its positive association with TSH—a pattern distinct from other PFASs—suggests unique hypothalamic-pituitary-thyroid axis stimulation (Zhou et al., 2025). Molecular docking analysis revealed that PFBS, despite being a shorter-chain alternative to legacy PFAS, can interfere with thyroid hormone synthesis and transport. These findings align with our random forest results showing PFBS as the most influential PFAS in the IPP group (mean %IncMSE 26.4%), and support the biological plausibility of PFBS-associated thyroid disruption in this vulnerable population. Negative associations were observed between PFHpA with T3 in the CPP cohort, and between PFHpA with T4 in the control group, while no significance in IPP group was observed. Researchers considered PFHpA and other PFASs but found no association between PFHpA and THs levels in most studies in maternal and cord blood (Reardon et al., 2019; Itoh et al., 2019; Aimuzi et al., 2020). Studies in the field of epidemiology have examined the link between prenatal PFAS exposure and changes in thyroid function for both mothers and their infants. Furthermore, numerous studies released prior to the identification of PFOA and PFOS have suggested that exposure to various long-chain PFAS might influence THs levels in both mothers and newborns (Coperchini et al., 2020).

We observed that negative relationships were easier to show between higher PFBS and PFOA concentrations and THs. While in recent studies, relationships between PFASs exposure and THs are concentration-dependent and non-linear. Among all the PFASs, PFOA has been most consistently associated with TH. A recent meta-analysis by Zhang et al. (2025) synthesized evidence from 20 studies on PFAS-thyroid associations in men and non-pregnant women, finding that higher PFOA concentrations were positively associated with free triiodothyronine (FT3) levels (Zhang et al., 2025). However, this association was not observed in smaller studies, indicating potential variability due to statistical power or population differences. Our study extends this evidence to a novel pediatric population (girls with CPP/IPP), where we observed an inverse association between PFOA and total T4, suggesting that age and clinical phenotype may modify PFAS-thyroid relationships. As a shorter-chain PFAS, PFBS presented as a PFOA alternative, was detected that lower PFBS concentrations were observed with abnormal or low T4 levels, suggesting an opposite relationship with thyroid function (Rosen Vollmar et al., 2023).

Random forest analysis results validated and extended the main findings from traditional regression. First, the feature importance ranking confirmed that PFOA and PFBS were the key PFASs affecting thyroid function. Second, random forest revealed inter-group heterogeneity in the effects of PFASs on THs: in the IPP group, the importance of PFBS was significantly elevated, and PFOS also showed strong importance, suggesting that IPP girls may be more sensitive to specific PFASs. Third, random forest identified the specific effects of long-chain PFASs (PFNA, PFUdA) on rT3, which might have been masked by multicollinearity in traditional regression. A potentially novel finding in our study was the inverse association between long-chain PFASs (PFNA, PFUdA) and rT3 levels specifically in the IPP group, which was not observed in the CPP or control groups. rT3 is generated primarily by the action of type 3 deiodinase (DIO3), which converts T4 to rT3 and T3 to T2, thereby inactivating thyroid hormones. One plausible mechanism is that long-chain PFASs may upregulate DIO3 expression or activity, leading to increased rT3 clearance or altered rT3 metabolism. Recent evidence has shown that PFAS exposure can impact thyroid hormone homeostasis through epigenetic modifications of thyroid hormone-related genes. A study demonstrated that maternal PFAS concentrations were associated with placental DNA methylation of DIO3 and other thyroid hormone-related genes, suggesting an epigenetic mechanism (Xie et al., 2025). Furthermore, Li et al. found that PFOS influences thyroid function through multiple signaling pathways including PPAR and AMPK pathways, which may indirectly affect deiodinase expression (Li et al., 2025). The fact that this association was specific to the IPP group is intriguing. IPP girls have isolated breast development or pubic hair without full activation of the hypothalamic-pituitary-gonadal axis, which may represent a state of partial or altered neuroendocrine maturation. It is possible that this developmental state confers differential sensitivity in peripheral thyroid hormone metabolism. Alternatively, unmeasured factors specific to the IPP phenotype, such as subtle differences in adiposity or steroid hormone profiles, may interact with PFASs to influence rT3 metabolism. Further experimental studies, including *in vitro* DIO3 activity assays and animal models, are needed to test the hypothesis that long-chain PFASs directly affect rT3 metabolism. Finally, prediction performance analysis showed that PFASs could explain 28%–35% of T4 variance, but had weaker explanatory power for T3 and rT3, suggesting that T4 may be the primary target of PFASs’ thyroid-disrupting effects. In this study, we observed discrepancies in the associations between PFASs and THs in different groups. The variations in PFAS–thyroid associations across different cohorts are notable, but understanding the underlying mechanisms requires further research. Some researchers propose that the rise in thyroxine-binding globulin production due to estrogen affects the assessment of thyroid function in the bloodstream (Tahboub and Arafah, 2009). This finding might explain the different associations between girls with and without sexual development.

This study had several strengths and limitations. This study contributes novel data on the associations between PFASs and thyroid hormones in girls with CPP and IPP. Its distinctive features include the inclusion of the IPP group as a separate clinical phenotype and the use of random forest machine learning to identify PFOA and PFBS as the PFASs most strongly associated with thyroid hormone alterations. Notably, our study was not designed to prove or disprove the direct role of PFASs in THs in girls with CPP. This observational study was designed to capitalize on the availability of structured clinical information on a reasonably large cohort of individuals in a wide spectrum of CPP and IPP. Regarding this study’s limitations, first, we did not measure TPO antibodies, which are markers of autoimmune hypothyroidism, and children with autoimmune thyroid impairment have reduced regulation of THs (Webster et al., 2016). Moreover, we did not investigate TSH, fT4, and FT3 levels. TSH is secreted by the pituitary gland, which is a crucial factor in the feedback between T4, T3, and the hypothalamus–pituitary–thyroid axis, and is more sensitive than T3 and T4 for measuring the thyroid function of individuals. Notably, Free THs provide a better reflection of THs in use than total THs, which can be interfered with by thyroid-binding globulin. Potential confounders such as family income, dietary iodine intake, and residential area were not included in this study, which may influence thyroid function. Future studies should consider these factors. Although we tested for interactions with obesity and maternal age at menarche and found no significant effect modification, the sample size in the obese subgroup (n = 93) was relatively small, which may have limited our power to detect true interactions. Future studies with larger sample sizes across different BMI categories are needed to more definitively assess potential effect modification. Another important limitation of this study is the absence of TSH, FT3, and FT4 measurements. These indicators, particularly TSH, are key to assessing the feedback status of the hypothalamic–pituitary–thyroid axis and are more sensitive than total T3/T4 for evaluating thyroid function. Future studies should include TSH and free thyroid hormones to better elucidate the effects of PFASs on the thyroid axis. An additional limitation is that a portion of the study period (January 2021 to January 2022) overlapped with COVID-19-related lockdowns and lifestyle disruptions in Shanghai. Changes in dietary patterns (e.g., increased consumption of packaged or delivered foods) and time spent indoors during this period may have influenced both PFAS exposure routes and thyroid hormone homeostasis. While we could not directly quantify these effects due to lack of related data, readers should be aware that the observed associations might be influenced by pandemic-related behavioral changes. Future studies should account for such large-scale societal disruptions when interpreting exposure-outcome associations.

## 5 Conclusion

In summary, we measured serum concentrations of 25 PFASs in 226 girls with CPP and 401 healthy girls. The integration of random forest analysis with traditional regression represents a methodological strength, enabling identification of PFOA and PFBS as the PFASs most strongly associated with thyroid hormone alterations in this study. These findings have important public health implications, suggesting that girls with IPP may particularly sensitive population for PFAS-associated endocrine alterations. Given the widespread exposure of children to PFASs and the essential role of THs in growth and neurodevelopment, these findings support the need for further hypothesis-driven research–including prospective cohort studies and mechanistic investigations in animal models–to test whether PFASs contribute to pubertal and thyroid dysfunction. Such research would provide a stronger evidence base for informing future regulatory policies aimed at protecting children’s health.

## Data Availability

The raw data supporting the conclusions of this article will be made available by the authors, without undue reservation.
