# Supplementary material for: Differential correlations of serum PFASs with thyroid hormones in girls with central and incomplete precocious puberty: a machine learning analysis
Source: Front Mol Biosci. 2026 Jul 22;13:1884470. doi: 10.3389/fmolb.2026.1884470 (PMC13437386; doi:10.3389/fmolb.2026.1884470)
Supplement: Supplementary file 1 [file Table1.docx]

Supplementary Table 1. The raw P-values and FDR-adjusted q-values of 12 PFASs across T3, T4, and rT3 in control group

| Analytes | T3 (raw P) | T3 (FDR Q) | T4 (raw P) | T4 (FDR Q) | rT3 (raw P) | rT3 (FDR Q) |
| --- | --- | --- | --- | --- | --- | --- |
| PFHpA | 0.906 | 0.906 | 0.049 | 0.098 | 0.487 | 0.584 |
| PFOA | 0.279 | 0.387 | 0.026 | 0.078 | 0.158 | 0.258 |
| PFNA | 0.228 | 0.387 | 0.673 | 0.733 | 0.541 | 0.584 |
| PFDA | 0.53 | 0.579 | 0.736 | 0.766 | 0.584 | 0.584 |
| PFUnDA | 0.975 | 0.975 | 0.826 | 0.826 | 0.327 | 0.441 |
| PFDoDA | 0.952 | 0.975 | 0.978 | 0.978 | 0.481 | 0.584 |
| PFBS | 0.179 | 0.322 | 0 | 0 | 0.939 | 0.959 |
| PFHxS | 0.149 | 0.322 | 0.174 | 0.29 | 0.351 | 0.451 |
| PFHpS | 0.936 | 0.955 | 0.936 | 0.936 | 0.343 | 0.451 |
| PFOS | 0.318 | 0.387 | 0.444 | 0.519 | 0.146 | 0.258 |
| 6:2Cl-PFESA | 0.82 | 0.861 | 0.144 | 0.259 | 0.695 | 0.725 |
| 8:2Cl-PFESA | 0.446 | 0.535 | 0.534 | 0.584 | 0.681 | 0.725 |

P values were adjusted for multiple comparisons using the Benjamini–Hochberg false discovery rate (FDR) method with a threshold of P < 0.20. Raw P values are shown for transparency, and FDR-adjusted P values are provided in the table or indicated by footnotes.
