# Supplementary material for: Differential correlations of serum PFASs with thyroid hormones in girls with central and incomplete precocious puberty: a machine learning analysis
Source: Front Mol Biosci. 2026 Jul 22;13:1884470. doi: 10.3389/fmolb.2026.1884470 (PMC13437386; doi:10.3389/fmolb.2026.1884470)
Supplement: Supplementary file 2 [file Table2.docx]

Supplementary Table 2. The raw P-values and FDR-adjusted q-values of 12 PFASs across T3, T4, and rT3 in CPP group

| Analytes | T3 (raw P) | T3 (FDR Q) | T4 (raw P) | T4 (FDR Q) | rT3 (raw P) | rT3 (FDR Q) |
| --- | --- | --- | --- | --- | --- | --- |
| PFHpA | 0.022 | 0.079 | 0.573 | 0.622 | 0.136 | 0.225 |
| PFOA | 0.641 | 0.706 | 0.022 | 0.079 | 0.367 | 0.441 |
| PFNA | 0.2 | 0.3 | 0.282 | 0.363 | 0.162 | 0.243 |
| PFDA | 0.225 | 0.3 | 0.413 | 0.478 | 0.165 | 0.243 |
| PFUnDA | 0.115 | 0.23 | 0.652 | 0.684 | 0.167 | 0.243 |
| PFDoDA | 0.243 | 0.3 | 0.491 | 0.552 | 0.26 | 0.338 |
| PFBS | 0.164 | 0.246 | 0.016 | 0.072 | 0.108 | 0.216 |
| PFHxS | 0.578 | 0.622 | 0.029 | 0.079 | 0.521 | 0.57 |
| PFHpS | 0.155 | 0.246 | 0.428 | 0.489 | 0.901 | 0.922 |
| PFOS | 0.227 | 0.3 | 0.61 | 0.658 | 0.996 | 0.996 |
| 6:2Cl-PFESA | 0.952 | 0.952 | 0.196 | 0.294 | 0.744 | 0.775 |
| 8:2Cl-PFESA | 0.248 | 0.3 | 0.156 | 0.243 | 0.43 | 0.49 |

P values were adjusted for multiple comparisons using the Benjamini–Hochberg false discovery rate (FDR) method with a threshold of P < 0.20. Raw P values are shown for transparency, and FDR-adjusted P values are provided in the table or indicated by footnotes.
