# Supplementary material for: Differential correlations of serum PFASs with thyroid hormones in girls with central and incomplete precocious puberty: a machine learning analysis
Source: Front Mol Biosci. 2026 Jul 22;13:1884470. doi: 10.3389/fmolb.2026.1884470 (PMC13437386; doi:10.3389/fmolb.2026.1884470)
Supplement: Supplementary file 3 [file Table3.docx]

Supplementary Table 3. The raw P-values and FDR-adjusted q-values of 12 PFASs across T3, T4, and rT3 in IPP group

| Analytes | T3 (raw P) | T3 (FDR Q) | T4 (raw P) | T4 (FDR Q) | rT3 (raw P) | rT3 (FDR Q) |
| --- | --- | --- | --- | --- | --- | --- |
| PFHpA | 0.386 | 0.463 | 0.317 | 0.399 | 0.176 | 0.317 |
| PFOA | 0.867 | 0.892 | 0.168 | 0.302 | 0.047 | 0.141 |
| PFNA | 0.602 | 0.662 | 0.165 | 0.302 | 0.04 | 0.141 |
| PFDA | 0.78 | 0.835 | 0.188 | 0.302 | 0.089 | 0.2 |
| PFUnDA | 0.654 | 0.7 | 0.261 | 0.36 | 0.028 | 0.141 |
| PFDoDA | 0.889 | 0.892 | 0.069 | 0.18 | 0.071 | 0.185 |
| PFBS | 0 | 0 | 0 | 0 | 0.056 | 0.144 |
| PFHxS | 0.834 | 0.877 | 0.127 | 0.286 | 0.551 | 0.62 |
| PFHpS | 0.912 | 0.912 | 0.112 | 0.269 | 0.137 | 0.271 |
| PFOS | 0.378 | 0.463 | 0.002 | 0.012 | 0.792 | 0.843 |
| 6:2Cl-PFESA | 0.552 | 0.619 | 0.17 | 0.302 | 0.324 | 0.404 |
| 8:2Cl-PFESA | 0.676 | 0.721 | 0.113 | 0.269 | 0.669 | 0.722 |

P values were adjusted for multiple comparisons using the Benjamini–Hochberg false discovery rate (FDR) method with a threshold of P < 0.20. Raw P values are shown for transparency, and FDR-adjusted P values are provided in the table or indicated by footnotes.
